# High baseline prevalence of atopic comorbidities and medication use in children treated with allergy immunotherapy in the REAl-world effeCtiveness in allergy immunoTherapy (REACT) study

**DOI:** 10.3389/fped.2023.1136942

**Published:** 2023-03-28

**Authors:** Benedikt Fritszching, Celeste Porsbjerg, Sarah Buchs, Julie Rask Larsen, Nick Freemantle, Marco Contoli

**Affiliations:** ^1^ Paediatric Pulmonology and Allergy, Children’s Doctor Service, Heidelberg, Germany; ^2^ Department of Respiratory Medicine, Bispebjerg Hospital, University of Copenhagen, Copenhagen, Denmark; ^3^ Global Market Access, ALK-Abelló, Hørsholm, Denmark; ^4^ Global Medical Affairs, ALK-Abelló, Hørsholm, Denmark; ^5^ Institute of Clinical Trials and Methodology, University College London, London, United Kingdom; ^6^ Respiratory Section, Department of Translational Medicine, University of Ferrara, Ferrara, Italy

**Keywords:** Allergic rhinitis, allergy immunotherapy, asthma, atopic comorbidities, atopic dermatitis, disease burden, pediatric, real-world evidence (RWE)

## Abstract

**Background:**

Respiratory allergy, commonly manifesting as allergic rhinitis (AR) and asthma, is a chronic progressive disease that frequently starts in childhood. Allergy immunotherapy (AIT) is the only causal treatment for respiratory allergy with the potential to modify the underlying cause of allergy and, ultimately, prevent disease progression. This analysis aimed to determine if AIT is received sufficiently early to halt the progression of allergic disease, by characterizing the burden and progression of disease in children prior to AIT initiation in real-life clinical practice.

**Methods:**

The REAl-world effeCtiveness in allergy immunoTherapy (REACT) study was a large retrospective cohort study using German claims data between 2007 and 2017. Characteristics of two pre-defined AIT age cohorts from the REACT study – children (aged <18 years) and adults (aged ≥18 years) – were evaluated during the 1-year period before the first AIT prescription. For comparison, a control group of all subjects with a confirmed diagnosis of AR and without prescriptions for AIT was included. Burden of disease was assessed using diagnostic codes for atopic comorbidities [e.g., atopic dermatitis (AD), asthma, and acute allergic conjunctivitis] and non-atopic comorbidities (e.g., migraine, headache); medication use, recorded as prescriptions for symptom-relieving AR medication and reliever/controller medication for asthma, was also assessed. Data were analyzed descriptively, using summary statistics.

**Results:**

Both children (*n* = 11,036) and adults (*n* = 30,037) showed a higher prevalence of atopic comorbidities and a greater drug burden prior to AIT initiation compared to AR patients not treated with AIT (*n* = 1,003,332). In the two age-specific AIT cohorts, children consistently showed the highest prevalence of atopic comorbidities compared to adults (AIT children, AIT adults – asthma: 41.4%, 34.5%; AD: 19.9%, 10.2%; acute allergic conjunctivitis: 13.6%, 10.2%). Generally, prescriptions per year for symptom-relieving AR and asthma treatments were also higher for children initiating AIT vs. adults (AIT children, AIT adults – AR prescriptions per subject: 1.72, 0.73; asthma prescriptions per subject: 1.42, 0.79).

**Conclusions:**

Children with AR who are offered AIT in real-life show considerable disease burden prior to initiation. As AIT may alleviate the burden and halt the progression of allergic disease, considering AIT earlier in the disease course may be warranted.

## 1. Introduction

Allergy is caused by an abnormal reaction of the immune system to otherwise harmless allergens (1). Allergies are highly prevalent; up to 40% of children and approximately 10%–30% of adults are affected by some form of allergy, and respiratory allergy is most common (1). Evidence suggests that the prevalence of respiratory allergy is increasing in many countries worldwide (2–4); although, recent trends show variability between regions (5). The most common manifestations of respiratory allergy are allergic rhinitis (AR) and allergic asthma (1, 6), which are also key components of the atopic triad, together with atopic dermatitis (AD) (7).

The “allergic march” describes the progression of allergic diseases from AD and food allergy in infancy to the gradual development of AR and asthma in childhood (8) – albeit with substantial heterogeneity in the clinical course between individuals. The progressive nature of allergic disease has been demonstrated in several birth cohort studies, where it has been reported that the risk of developing AR is increased by up to 5-fold in children with AD and the risk of developing asthma is increased by up to 7-fold in children with AR (9–11). Consequently, clinical guidelines [Allergic Rhinitis and its Impact on Asthma (ARIA) and European Academy of Allergy and Clinical Immunology (EAACI)] recommend considering AR as a risk factor for developing asthma (12, 13). Indeed, AR and asthma are considered to be manifestations of one syndrome in different parts of the respiratory tract (upper and lower airways) (14), such that improving the control of one condition has benefits for the other (15).

Allergic disease frequently starts in childhood and often has a negative impact on the quality of life of affected children and their families (16). Symptoms of AR can affect a child's quality of life through disturbances in sleep (17, 18), memory impairment (19), impaired performance in school (20), and restrictions in social activities (17). Children with symptomatic AR may also be more susceptible to complications of the upper airway, resulting in sinus problems, pain, and sometimes surgery, than children without AR (17). Despite this burden, AR is often underestimated in children and is trivialized as a disease of lesser importance. However, research into AR in children is increasing, i.e., non-invasive measures, such as rhinomanometry and the measurement of fractional excretion of nitric oxide (FeNO) are currently under evaluation to determine if they will prove useful in clinical decision making (21–23); modulation of the microbiome for AR prevention is also under investigation (24).

Often, children suffer from other manifestations of the underlying allergic disease, in addition to AR. Multimorbidity of allergic diseases in children may result in further worsening of their quality of life; children with symptoms of more than one allergic disease (eczema, asthma, or AR) have shown a greater level of emotional and hyperactive problems than children without atopic disease (25). Aside from the physical and emotional impacts of allergic disease on children, atopic conditions can have a considerable economic impact, particularly when they are severe (26–28). The coexistence of multiple allergic diseases, such as asthma and AD, can further increase healthcare costs (27). Upon entry into the workplace, allergic conditions can contribute to lost productivity, with subsequent socio-economic consequences (29, 30). AD commonly coexists with AR (31), and has a negative effect on patients, caregivers, and society through sleep disturbances, a negative impact on social life, and lost productivity (32). Children with AD are often avoided by their peers (33), and parents actively withdraw from social interaction to avoid discussing their child's condition (34). AD can impair the psychological well-being of affected children (33), as well as their ability to learn (35). Furthermore, hospitalization rates are higher among children with AD who develop infections vs. those who do not (36). Consequently, AD remains an extremely disabling condition, especially in the pediatric population. Biological drugs have shown promise as a treatment for the more severe forms of AD (37), whereas other drugs, such as probiotics, need further research before routine use can be recommended (38). Asthma is another common comorbidity of AR (39–41), and has been associated with limitations in the amount and type of physical activity that affected children can participate in, which can have a subsequent negative impact on their ability to socialize with peers, and to build relationships; it can also affect their self-esteem (42). Asthma is also one of the most common causes of pediatric hospital admissions (43, 44). In children with allergic asthma, real-life allergen exposure combined with natural virus exposure has been shown to increase the risk of hospitalization due to an acute asthma exacerbation (43).

Allergy immunotherapy (AIT) is, currently, the only causal treatment option for respiratory allergy (13, 45). It has been suggested that a window of opportunity for halting the allergic march and preventing disease progression, e.g., from AR to asthma (and new allergic sensitizations) may exist, primarily, in young children with mild allergy symptoms and a low level of allergic sensitization (46). Consequently, it is important to characterize the burden and progression of allergic disease in children prior to initiation of AIT in real-life clinical practice. The recently published REAl-world effeCtiveness in allergy immunoTherapy (REACT) study was a large, retrospective, and propensity score matched cohort study conducted in Germany, which demonstrated the real-world, long-term effectiveness of AIT for the treatment of AR and asthma in subjects with prescriptions for AIT vs. matched AR controls without AIT prescriptions (47). Before matching, the overall AIT group of subjects was found to be younger, with more atopic comorbidities and a greater drug burden, compared to those with a diagnosis of AR who did not have prescriptions for AIT indicating that, in real-life clinical practice, AIT is mainly offered to AR subjects who already have a substantial burden of disease (47).

The aim of the current analysis was to determine if AR subjects receive AIT sufficiently early to potentially halt the progression of allergic disease. This was explored by characterizing the burden and progression of allergic disease in children prior to initiation of AIT in real-life clinical practice, using data from the REACT study.

## 2. Materials and methods

**Figure 1:**
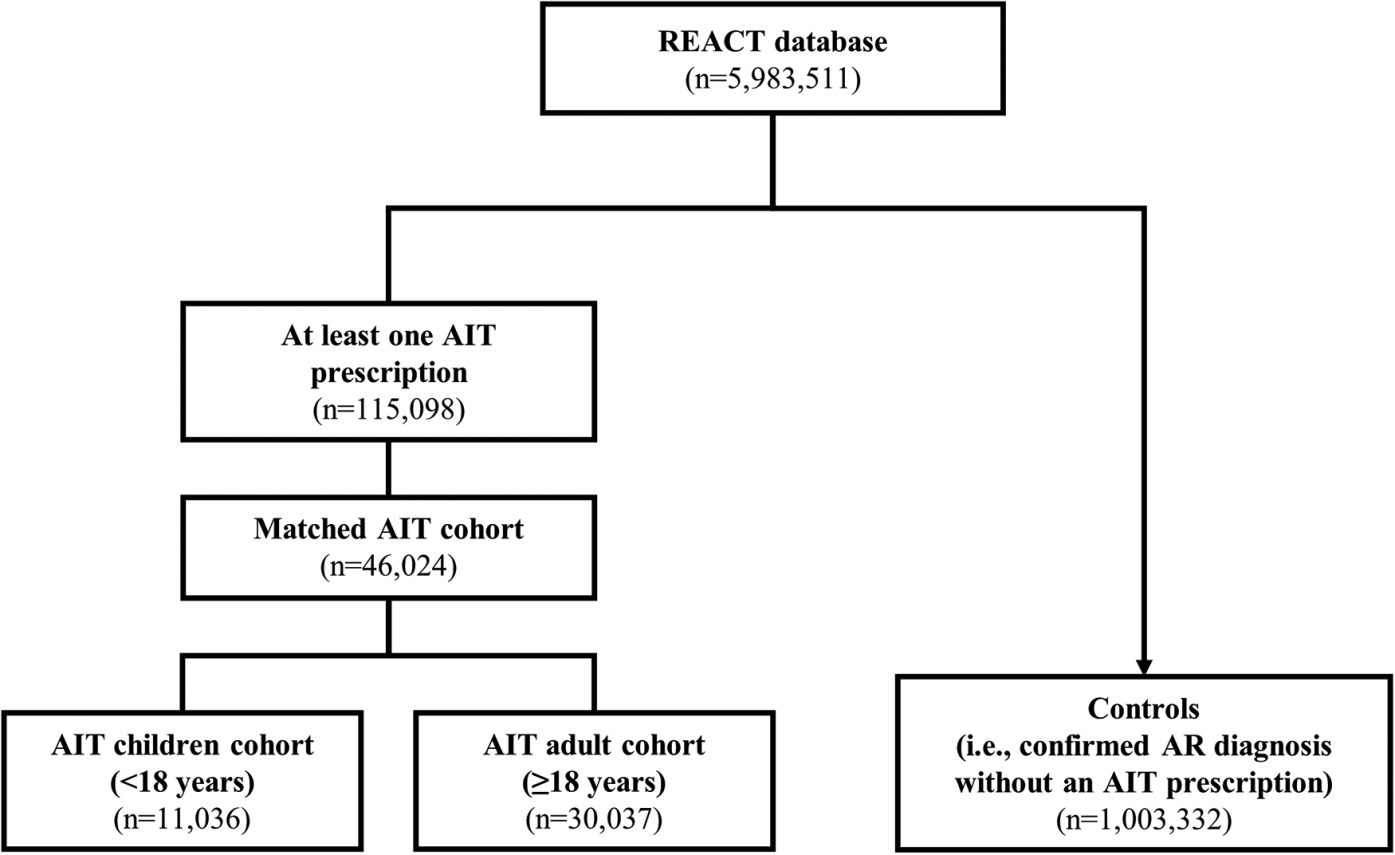
Subject selection in the main REACT study (2007–2017). Data are adapted from the main publication (47). The AIT age cohorts were formed by re-matching subjects from the main cohort, by age. AIT subjects who could not find a match were excluded from the cohorts. In addition to the children and adults in the AIT group, the entire group of subjects diagnosed with AR and without prescriptions for AIT were included as controls. AIT, allergy immunotherapy; AR, allergic rhinitis; REACT, REAl-world effeCtiveness in allergy immunoTherapy.

The REACT study (NCT04125888) was a retrospective cohort study that evaluated claims data from a German health insurance database [Betriebkrankenkasse (BKK)], which included data for approximately 5.9 million individuals (47). The REACT study methodology and findings have been published separately (47, 48). Briefly, anonymized insurance claims data for the study period (1 January 2007 to 31 December 2017) were reviewed, and subjects with a confirmed diagnosis of AR according to International Statistical Classification of Diseases and Related Health Problems 10th Revision (ICD-10) criteria (with or without asthma) were identified for evaluation (47). More than 1 million individuals in the database (18.7%) had a confirmed diagnosis of AR, but only 10.3% of these received at least one AIT prescription (Figure 1) (47). Subjects were eligible for the study if they had received at least two prescriptions of the same AIT during the first year (47). Subjects with a prescription for venom AIT and subjects with <12 months of follow-up before and after the index date (i.e., the date of the first AIT prescription) were excluded; thereafter, subjects who had received AIT and were successfully matched with a control (i.e., a non-AIT AR subject) were enrolled in the study (Figure 1) (47). From the overall AIT cohort, two pre-defined AIT cohorts according to age at the start of AIT were formed by re-matching AIT and non-AIT AR subjects: children (aged <18 years; *n* = 11,036) and adults (aged ≥18 years; *n* = 30,037) (Figure 1) (47). For comparison, the entire group of AR subjects without AIT prescriptions (*n* = 1,003,332) identified from the BKK database was included as controls in this analysis.

The current analysis used demographic data from the 1-year period before the first prescription of AIT (index date) for the pre-defined AIT age cohorts – children and adults – to characterize the burden of respiratory allergy. The demographic data were compared with a control group consisting of all subjects with a confirmed diagnosis of AR, but without AIT prescriptions. The burden of disease was assessed in three ways: (1) the proportion of subjects with confirmed ICD-10 diagnostic codes for atopic comorbidities (asthma, AD, and acute allergic conjunctivitis); (2) the proportion of subjects with any comorbidity affecting the lower/upper airways, eyes, skin, gastrointestinal tract, sleep, or the central nervous system; (3) the use of symptom-relieving medication for AR [all prescriptions and, separately, prescriptions for antihistamines, and intranasal corticosteroids (INCSs)], and for asthma [all prescriptions and, separately, prescriptions for short-acting beta agonists (SABAs), inhaled corticosteroids (ICSs), and ICSs + long-acting beta agonists (LABAs)]. The safety of AIT was assessed by the incidence of anaphylaxis related to AIT initiation, which was defined as the ICD-10 diagnostic code for anaphylactic shock (T78.2, T80.5, T88.6) within 2 days of the index date (47). Data were analyzed descriptively, using summary statistics.

## 3. Results

As previously reported, the REACT AIT age cohorts consisted of 11,036 children and 30,037 adults (Figure 1) (47). Table 1 presents the key characteristics of children and adults in the AIT group. The subgroup of “children” consisted of equal proportions of children (aged 0–11 years; 51.0%) and adolescents (aged 12–17 years; 49.0%). There was a higher proportion of males among children than among adults, and fewer ambulatory care visits were recorded for children than for adults. The duration of AIT was similar between children and adults, as was the incidence of anaphylactic shock around the time of AIT initiation.

**Table 1:** Key characteristics of children and adults in the AIT group.

Characteristic	AIT children cohort (<18 years)	AIT adult cohort (≥18 years)
	(*n* = 11,036)	(*n* = 30,037)
Age (years), median (IQR)	11 (9–14)	37 (28–46)
Age group, *n* (%)		
Children (0–11 years)	5,623 (51.0)	–
Adolescents (12–17 years)	5,413 (49.0)	–
Adults (≥18 years)	–	30,037 (100)
Males, *n* (%)	6,855 (62.1)	14,626 (48.7)
Health resource utilization, mean number per subject (SD)		
Ambulatory care (visits)	12.3 (8.1)	17.0 (13.4)
Hospitalizations (visits)	0.2 (0.7)	0.2 (0.6)
AIT treatment, *n* (%)		
Single allergen treatment	10,757 (97.5)	28,886 (96.2)
Duration of AIT treatment, mean (SD)		
Days on index AIT^a^	559.1 (271.2)	543.3 (288.6)
Days on any AIT	844.9 (453.1)	814.9 (478.6)
Anaphylactic shock at AIT initiation, *n* (%)	8 (0.07)	22 (0.07)

Data presented are for the pre-defined cohorts according to age at the start of AIT. Demographic data for all AR subjects without AIT prescriptions (i.e., controls), identified from the BKK database, were published previously (47).

^a^ Index AIT was defined as the first AIT that was prescribed during the study period (excluding venom AIT). AIT, allergy immunotherapy; AR, allergic rhinitis; BKK, Betriebkrankenkasse; IQR, interquartile range; SD, standard deviation.

### 3.1. Key atopic comorbidities

**Figure 2:**
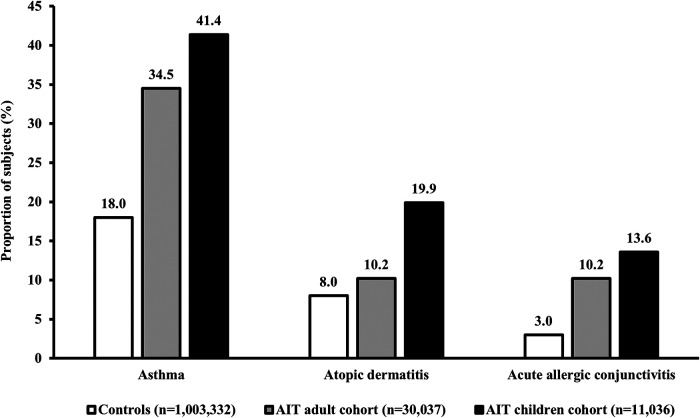
Prevalence of key atopic comorbidities in subjects with AR. Data presented are from the 1-year period before the index date (i.e., the first prescription for AIT during the study period) for children and adults in the AIT group, and for control subjects (i.e., all AR subjects without prescriptions for AIT) (published previously) (47). Asthma was defined based on the ICD-10 diagnostic code J45.x, or J46 and/or at least two prescriptions of SABA/ICS within an index year. AD and acute allergic conjunctivitis were defined according to the ICD-10 diagnostic codes L20–L20.9 and H10.1, respectively. AIT, allergy immunotherapy; AR, allergic rhinitis; ICD-10, International Statistical Classification of Diseases and Related Health Problems 10th Revision; ICS, inhaled corticosteroid; SABA, short-acting beta agonist.

The prevalence of atopic comorbidities (asthma, AD, and acute allergic conjunctivitis) during the 1-year period before the first prescription for AIT was higher for both AIT cohorts (children and adults) than for the control cohort (Figure 2). Among AIT subjects, children consistently showed the highest prevalence of comorbidities compared with adults across the three separate diseases (Figure 2).

### 3.2. All comorbidities

**Figure 3:**
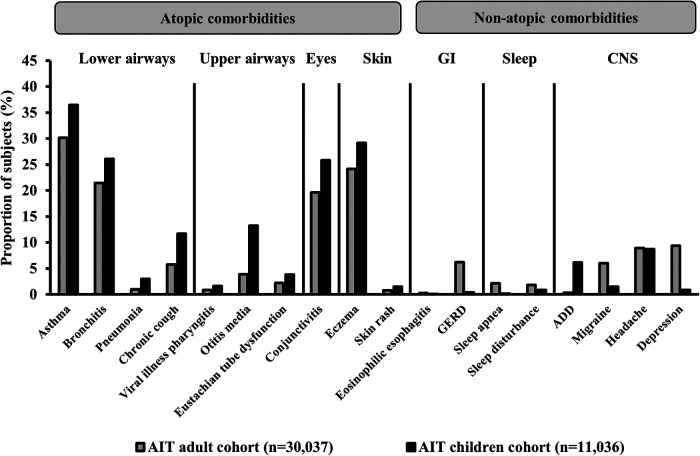
Prevalence of comorbidities in subjects with AR who had received AIT prescriptions. Data presented are from the 1-year period before the index date (i.e., the first prescription for AIT during the study period) for subjects in the AIT group. ICD-10 diagnostic codes – asthma: J45.x, J46; bronchitis: J20.x, J40.x–J42, J44.x; pneumonia: J12.x–J18.x; chronic cough: R05; viral illness pharyngitis: J10.1, B00.2, B08.5, B08.8, B27.x; otitis media: H65.x–H67.x; Eustachian tube dysfunction: H68.1–H69.x; conjunctivitis: H10.x; eczema: L20.x–L30.x, L50.x–L54.x; skin rash: R21; eosinophilic esophagitis: K20; GERD: K21.x; sleep apnea: G47.3; sleep disturbance: G47.0–G47.2, F51.x; ADD: F90.x; migraine: G43.x; headache: R51, G44.x; depression: F32.x–F33.x. ADD, attention deficit disorder; AIT, allergy immunotherapy; CNS, central nervous system; GERD, gastroesophageal reflux disorder; GI, gastrointestinal; ICD-10, International Statistical Classification of Diseases and Related Health Problems 10th Revision.

Generally, in the AIT group, the prevalence of all comorbidities during the 1-year period before the first prescription for AIT was higher for children than for adults (Figure 3). Conditions such as asthma, conjunctivitis, otitis media, and AD, which are typically associated with respiratory allergies and/or airway infection, occurred in a higher proportion of children than adults, whereas conditions such as gastroesophageal reflux disorder, migraine, and depression were more prevalent in adults than in children.

### 3.3. Use of medication for AR and asthma

**Figure 4:**
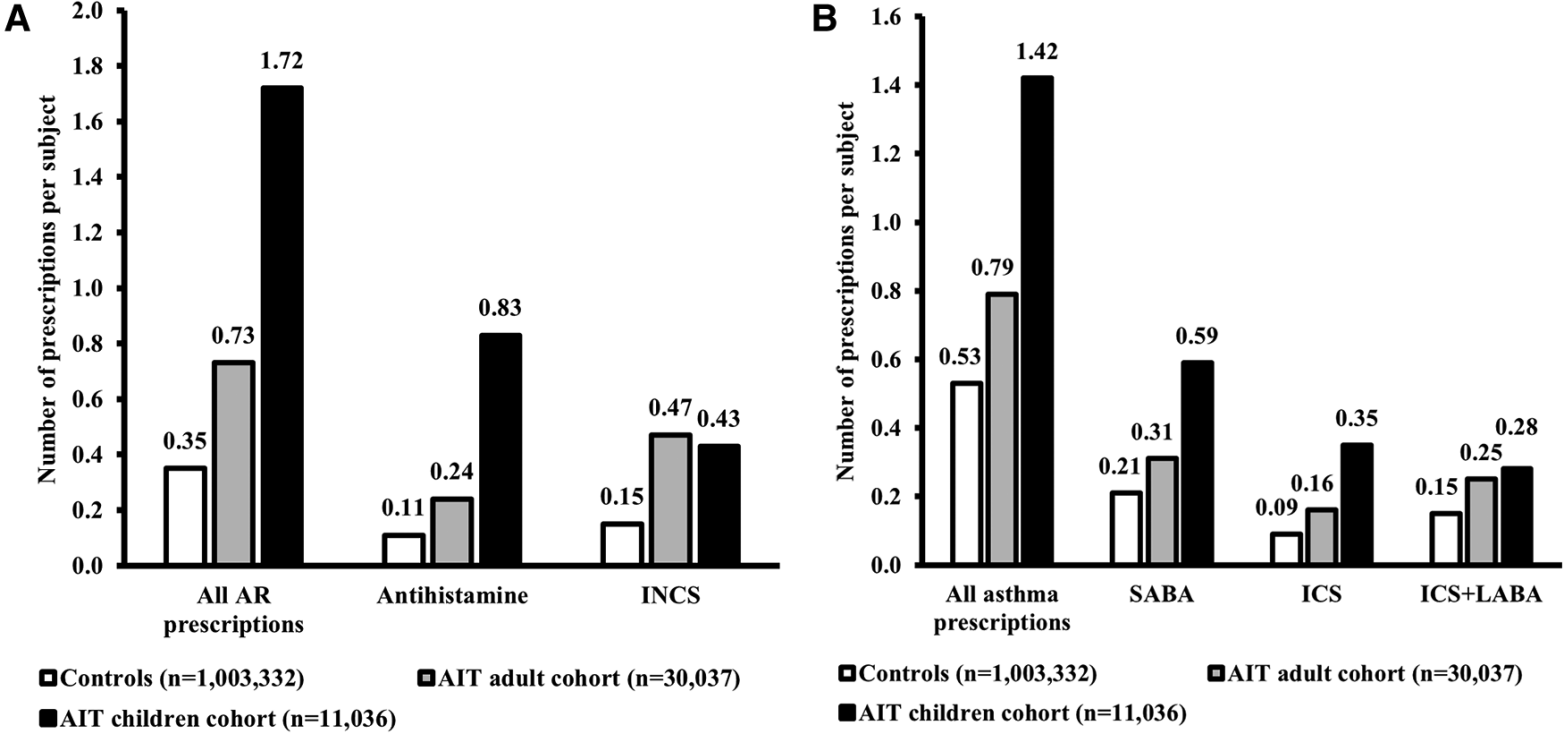
Medication use for AR (**A**) and asthma (**B**). Data presented are from the 1-year period before the index date (i.e., the first prescription for AIT during the study period) for children and adults in the AIT group, and for control subjects (i.e., all AR subjects without prescriptions for AIT) (47). The “All AR prescriptions” and “All asthma prescriptions” data for AR subjects without AIT have been published previously (47). AIT, allergy immunotherapy; AR, allergic rhinitis; ICS, inhaled corticosteroid; INCS, intranasal corticosteroid; LABA, long-acting beta agonist; SABA, short-acting beta agonist.

Generally, AR and asthma prescriptions per subject during the 1-year period before the index date were higher for children than adults in the AIT group and the control cohort (Figure 4). This effect was consistent across individual AR and asthma drug classes, with the exception of INCSs for AR, which showed a comparable number of prescriptions between children and adults of the AIT group (Figure 4).

## 4. Discussion

The findings of this analysis expand the learnings from the main REACT study by focusing on the burden of disease before AIT initiation (47). AR, defined using stringent and established ICD-10 criteria, was diagnosed in almost one fifth of individuals in the BKK database confirming that it is a highly prevalent disease. The disease burden associated with AR is high and is further exacerbated by the presence of concomitant comorbid conditions. Despite this considerable burden, the use of AIT is low (in the REACT study, 10.3% of subjects diagnosed with AR received prescriptions for AIT) and appears to be restricted to subjects with AR of greater severity (47). A previous study of claims data reported the use of AIT by 7% of AR subjects in Germany (49); a decade later, the findings of this analysis indicate a distinct lack of uptake in the use of AIT. This is consistent with the results of a large, multi-phase epidemiological study [der Studie zur Gesundheit von Kindern und Jugendlichen in Deutschland (KiGGS)], which was conducted in Germany between 2003 and 2017 (around the time of the REACT study) (50, 51). The study investigated AIT use in subjects aged 11–17 years with AR or AD who had received a positive allergy test (51). While the use of allergy testing may suggest that these children were receiving specialist care (unlike those in the REACT study), only 30% received AIT following this positive result (51). By examining the disease burden in children and adults with AR initiating treatment with AIT, the current analysis adds to our knowledge of the disease and shows that allergic disease is already relatively advanced when children initiate AIT in real-life clinical practice.

Overall, prior to AIT initiation, the proportion of subjects with key atopic comorbidities (asthma, AD, and/or acute allergic conjunctivitis) was higher, and the drug burden was greater, among children than adults. The findings regarding the burden of comorbidities are in line with the results from an observational cohort study in Spain (52). In the current analysis, children who initiated AIT also showed a higher prevalence of general comorbidities than adults, mainly driven by conditions related to allergy (e.g., asthma) and infection (e.g., otitis media), whereas adults were more prone to having non-allergic comorbidities (e.g., migraine). These observations align with the knowledge that respiratory allergy is more prevalent in children than in adults (53, 54), and that AR is associated with an increased risk of infection (55, 56). The negative effect of AR and asthma on sleep is well-known (6). In adults with perennial AR, sleep problems (difficulty falling asleep, poor sleep) are reported to have a negative impact on productivity and quality of life; the sleep problems and subsequent effects are more pronounced in individuals who also have a diagnosis of asthma (57). In the REACT study, sleep disorders were confirmed using formal ICD-10 diagnostic codes and were slightly more prevalent in adults than in children; this may have resulted in an underestimation of such comorbidities, particularly in children, as sleep problems may have been viewed as a symptom, rather than as an individual condition.

Generally, in the AIT group, children showed greater use of medication for AR and for asthma than adults. There could be many reasons for this observation, ranging from differences in compliance to differences in prescribing patterns between children and adults (financial incentives in Germany may have resulted in more medication prescriptions in children). It is possible that the medication use for AR may have been underestimated as the analysis did not account for over-the-counter AR treatments. However, the same overall signal was observed for asthma medications, which are available by prescription only. For asthma, ICS use was higher for children than for adults but, for AR, INCS use was similar between the two age groups. This observation is in line with the treatment guidelines in children – corticosteroids are recommended as first-line treatment for asthma, but as a second-line treatment option for AR (antihistamines are the recommended first-line option) (58, 59). Differences in the pattern of asthma and AR medication usage by age may reflect differences in physicians' perception of the severity of the two conditions.

In the current analysis, the proportion of males was higher among children than adults in the AIT group (62.1% and 48.7%, respectively). This gender distribution between child and adult populations was also observed in the aforementioned Spanish study (52). It is recognized that, in childhood, males present with allergies more often than females and that during the years of female sexual development, the influence of various factors (e.g., sex hormones, lifestyle, diet, and adherence to treatment) switches the predominance of allergic diseases towards females (60). However, the mechanisms underlying these gender-specific differences in the prevalence of AR require further investigation (60).

The duration of AIT treatment was similar in children and adults, which may suggest similar tolerability to treatment across both age groups. Evidence from real-world studies vary – Vogelberg et al. (2020) reported that children (aged 5–11 years) are more persistent with AIT than adolescents (aged 12–17 years) or adults (aged 18–50 years) (61), whereas Borg et al. (2021) reported that compliance to AIT was generally similar, regardless of age (62). From a tolerability perspective, the rate of anaphylactic shock around initiation of AIT was low in children and adults, and is in line with published literature supporting the safety of AIT in pediatric respiratory allergy (63, 64). In the current analysis, the subgroup of “children” comprised an equal proportion of children (aged 0–11 years; 51%) and adolescents (aged 12–17 years; 49%). The behavioral needs and challenges for adherence differ across the two age groups. As discussed in a recent EAACI guideline, specific education and involvement of adolescents and young adults is needed to achieve optimal adherence to treatment (65).

The recently published real-world VerSITA study shows that, in Germany, certain groups of people with respiratory diseases are underprivileged in the healthcare system regarding the optimal care of their disease (66). It appears that AIT is underutilized in individuals with AR, and has been for more than a decade. Based on the burden of respiratory allergy in children demonstrated in this real-world analysis, the use of AIT in this patient population is clinically relevant. The EAACI guidelines highlight the potential disease-modifying effect of AIT to prevent asthma (13), which is supported by the findings of the pediatric Preventive Allergy Treatment (PAT) and Grazax Asthma Prevention (GAP) trials (67–69). In addition, a meta-analysis of AIT trials indicated the benefit of AIT in preventing asthma in subjects aged <18 years (vs. ≥18 years) (70). Such evidence implies that AIT has the potential to prevent/halt the atopic march. Indeed, the European Forum for Research and Education in Allergy and Airways diseases (EUFOREA) state that, given the increased risk of developing allergic asthma in preschool and school-age children and the burden of the disease, AIT should be considered within 2 years of allergic symptoms commencing (59).

The current analysis shows that, in the real-world, children initiating AIT already carry a high burden of disease, which calls for greater awareness of modern and evidence-based AIT options among pediatricians and general practitioners. Although the impact of allergic disease in children appears to be greater than in adults, there are currently fewer evidence-based AIT treatments approved for paediatric use. To date, the efficacy and safety of SQ sublingual immunotherapy (SLIT)-tablets for the treatment of AR have been demonstrated in children and adolescents (aged 5–17 years) with AR triggered by grass or ragweed pollen with or without conjunctivitis (71, 72), and in adolescents (aged 12–17 years) with AR triggered by house dust mite (HDM) (73). Two large, phase 3 trials are ongoing to confirm the efficacy and safety of SQ tree and HDM SLIT-tablets in younger children with AR (tree, aged 5–17 years; HDM, aged 5–11 years).^a^^,^^b^ In addition to efficacy and safety, it may also be relevant to consider the optimal route of administration for children. According to a reflection paper from the European Medicines Agency, orodispersables are the preferred formulation for children aged 2–5 years, and are the dosage form of choice for children aged ≥6 years (74). Within AIT, the SQ SLIT-tablet provides an orally dispersible formulation of AIT.

The key strength of this analysis is the use of a large, unselected population of subjects with AR who had received prescriptions for AIT in real-world clinical practice. Although some eligibility criteria were applied in the REACT study, these were kept to a minimum. An additional strength of the analysis is that the subgroups and methodology were pre-defined in the main study protocol. The analysis was retrospective in nature (1 January 2007 to 31 December 2017) meaning that clinical management may have changed over time, and this is noted as a limitation. Although the use of pre-defined subgroups is a strength of the analysis, it may also be considered a limitation as the group of subjects in the AIT cohort was rather selective relative to the pool of all non-AIT AR subjects. An additional limitation of the analysis is that not all subjects with AIT prescriptions were included; only those subjects who were eligible and could be matched, were further characterized. However, due to the underutilization of AIT, most subjects in the AIT group could successfully be matched with a similar control, and the impact of selection is thought to be minor.

In conclusion, the findings of the current analysis emphasize the considerable burden of allergic disease, particularly among children who are diagnosed with AR and offered AIT treatment. Although there is a growing body of evidence from randomized controlled trials and real-life observational studies supporting the efficacy and safety of AIT treatment in children, the analysis shows the high disease burden in children who initiate AIT in clinical practice. This finding suggests that AIT is, currently, offered to a limited group of eligible children. Physicians could consider initiating AIT in a broader range of eligible children and, also, offer AIT earlier in the disease course, in order to ameliorate the burden of disease and, potentially, halt the disease progression.

## Data Availability

The datasets presented in this article are not readily available because data are owned by the BKK sickness fund, which provides access to anonymized data derived from routinely collected administrative claims data. These data can only be accessed by a permitted 3^rd^ party (Team Gesundheit, Gesellschaft für Gesundheitsmanagement GmbH, Essen, Germany) for research purposes. Requests to access the datasets should be directed to https://teamgesundheit.de/.

## Footnotes

^a^ https://www.clinicaltrials.gov/ct2/show/NCT04145219

^b^ https://www.clinicaltrials.gov/ct2/show/NCT04878354
